# Insights into the Antioxidant/Antiradical Effects and In Vitro Intestinal Permeation of Oleocanthal and Its Metabolites Tyrosol and Oleocanthalic Acid

**DOI:** 10.3390/molecules28135150

**Published:** 2023-06-30

**Authors:** Doretta Cuffaro, Diana Pinto, Ana Margarida Silva, Andrea Bertolini, Simone Bertini, Alessandro Saba, Marco Macchia, Francisca Rodrigues, Maria Digiacomo

**Affiliations:** 1 Department of Pharmacy, University of Pisa, 56126 Pisa, Italy; doretta.cuffaro@unipi.it (D.C.); simone.bertini@unipi.it (S.B.); marco.macchia@unipi.it (M.M.); 2 Interdepartmental Research Center “Nutraceuticals and Food for Health”, University of Pisa, 56100 Pisa, Italy; 3 REQUIMTE/LAQV, ISEP, Polytechnic Institute of Porto, Rua Dr. António Bernardino de Almeida, 4249-015 Porto, Portugal; diana.pinto@graq.isep.ipp.pt (D.P.); ana.silva@graq.isep.ipp.pt (A.M.S.); 4 Department of Surgery, Medical, Molecular and Critical Area Pathology, University of Pisa, 56126 Pisa, Italy; a.bertolini2@student.unisi.it (A.B.); alessandro.saba@unipi.it (A.S.)

**Keywords:** polyphenols, oleocanthal, tyrosol, oleocanthalic acid, antioxidant activity, metabolism, intestinal permeation

## Abstract

(1) Background: In recent years, numerous studies have highlighted the beneficial effects of extra virgin olive oil (EVOO) as an active ingredient against chronic diseases. The properties of EVOO are due to its peculiar composition, mainly to its rich content of polyphenols. In fact, polyphenols may contribute to counteract oxidative stress, which often accompanies chronic diseases. In this work, the antioxidant effects of high-value polyphenol oleocanthal (OC) and its main metabolites, tyrosol (Tyr) and oleocanthalic acid (OA), respectively, have been investigated along with their impact on cell viability. (2) Methods: OC, Tyr, and OA have been evaluated regarding antiradical properties in term of scavenging capacity towards biologically relevant reactive species, including O_2_^●−^, HOCl, and ROO^●^, as well as their antioxidant/antiradical capacity (FRAP, DPPH^●^, ABTS^●+^). Moreover, the ability to permeate the intestinal membrane was assessed by an intestinal co-culture model composed by Caco-2 and HT29-MTX cell lines. (3) Results: The capacity of OC and Tyr as radical oxygen species (ROS) scavengers, particularly regarding HOCl and O_2_^●−^, was clearly demonstrated. Furthermore, the ability to permeate the intestinal co-culture model was plainly proved by the good permeations (>50%) achieved by all compounds. (4) Conclusions: OC, OA, and Tyr revealed promising properties against oxidative diseases.

## 1. Introduction

The nutritional model called the “Mediterranean diet” is related with several benefits for health, as widely documented in the literature [1,2,3]. Its positive effects are principally linked to the consumption of extra virgin olive oil (EVOO) that represents the main dietary lipid source of Mediterranean diet. In recent years, numerous clinical trials have highlighted the beneficial effects of EVOO, including antioxidant, anti-inflammatory, anti-diabetic, and anticancer properties [4,5,6]. These activities are principally attributed to EVOO’s minor constituents such as phenolic compounds, particularly flavanols, lignans, and secoiridoids [7,8]. Among the latter, which are phenolic compounds particularly representative of plants belonging to the Oleaceae family, the scientific interest has been directed towards oleocanthal (OC), even though it is present in EVOO in low amounts. The first identification of OC is recognized to Montedoro, in 1993 [9]. Later, in 2005, Beauchamp et al. [10] correlated the OC with the typical throat irritant sensation of EVOO. Moreover, the authors demonstrated the anti-inflammatory properties of OC were comparable with the nonsteroidal anti-inflammatory drug (NSAID) ibuprofen. As inflammation can be associated with the development of numerous chronic diseases, such as neuroinflammation, cardiovascular, and cancer diseases, in recent years OC was extensively studied to evaluate its involvement in these conditions. Several studies reported the anticancer [11,12] and neuroprotective effects promoted by OC [13,14,15,16]. In particular, different human clinical trials demonstrated the protective effect of OC by in vitro assays in arthropathy [17] as well as by in vivo studies on platelet aggregation in cardiovascular diseases [5].

**Figure 1:**
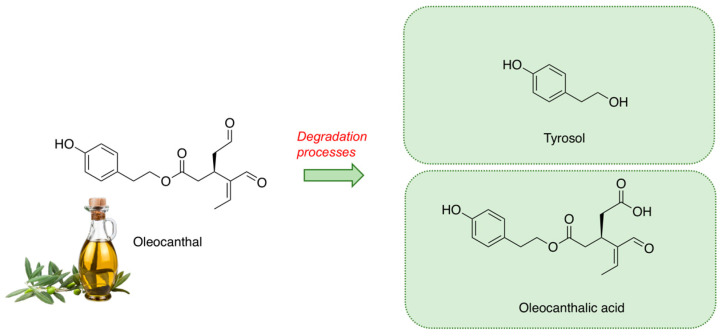
Chemical structure of OC and its metabolites Tyr and OA.

During EVOO storage, OC can undergo degradation processes, including hydrolytic and oxidative processes that are influenced by factors such as light, time, temperature, and oxygen [18,19,20] (Figure 1). Hydrolytic processes may cause the formation of tyrosol (Tyr), a simple phenol endowed with nutraceutical properties against insulin resistance and obesity and, in particular, coronary heart disease, chronic heart failure, hypertension, and atherosclerosis [21]. In fact, due to the hydrolytic processes, the high content of OC in fresh EVOO decreases during storage in sharp contrast with Tyr content, which increases. Furthermore, the oxidation of OC might lead to the formation of oleocanthalic acid (OA), a compound recently described by different authors [22,23]. In fresh EVOO, the content of OA is modest, increasing during long storage periods. To date, the potential nutraceutical properties of OA are poorly studied, with just one study demonstrating its neuroprotective capacity [22].

In our previous paper, the radical scavenging potential of OA against oxygen species was reported, evidencing its properties as an effective radical scavenger [23]. Therefore, it is imperative to understand the bioavailability of OC and its metabolites, OA and Tyr, to better evaluate their pro-health effects against oxidative diseases.

The main goal of this study was to assess the antioxidant/antiradical activity of OC and its metabolites (Tyr and OA) and to investigate the bioavailability of these nutraceutical polyphenols, estimating their paracellular permeation in an intestinal co-culture model composed of Caco-2 and HT29-MTX cell lines.

## 2. Results

### 2.1. Antiradical and Antioxidant Activities Evaluated by DPPH, ABTS, and FRAP Assays

The antiradical and antioxidant abilities of Tyr, OC, and OA were assessed using different in vitro studies to achieve more comprehensive data. Table 1 summarizes the DPPH, ABTS, and FRAP results obtained for each sample. Based on the FRAP results achieved, all compounds presented a good redox ability to reduce Fe^3+^ (Table 1). As it is possible to observe, OC and Tyr revealed a similar antioxidant profile, whereas OA was two times less potent. Concerning DPPH, a very commonly used assay to measure the antiradical effects, Tyr and OA presented a similar low scavenging capacity, reporting IC_50_ values higher than 1 mg/mL, while OC achieved an IC_50_ = 660 µg/mL. Regarding the ABTS^●+^ radical scavenging activity, all extracts led to an improved ability to inhibit the radical, with OC presenting an IC_50_ = 70.37 µg/mL, whereas its metabolites Tyr and OA each obtained an IC_50_ inferior to 10 µg/mL, being most effective.

### 2.2. Reactive Oxygen Species Scavenging Capacity

Table 2 summarizes the in vitro scavenging capacities of OC, Tyr, and OA against the different ROS tested. As far as we know, this is the first study that accomplished a comprehensive evaluation of the in vitro radicals scavenging activity of OC and Tyr. The results of the radical scavenging property of OA were recently reported by our research team [23]. Gallic acid and catechin were used as positive controls.

O_2_^●−^ is the first reactive species produced by oxygen reduction, being after converted into more powerful species, such as H_2_O_2_ and HOCl [24]. Concerning Tyr, like OA, it was not possible to calculate the IC_50_ due to the low scavenging efficiency. In this way, the result for Tyr was expressed as an inhibition percentage for the highest concentration tested (1000 µg/mL), achieving 17.05% of O_2_^●−^ inhibition, while for OA the value was 19.09% [23]. Significant differences (*p* < 0.05) were found between OC and Tyr, whereas the results obtained for gallic acid and catechin were not significantly different (*p* > 0.05).

HOCl is one of the most relevant reactive species formed during inflammatory processes. The reaction of H_2_O_2_ with chloride ions by myeloperoxidase produces HOCl in neutrophils [24]. The HOCl scavenging potential improved in the following order: OC (IC_50_ = 73.18 µg/mL) < OA (IC_50_ = 360.87 µg/mL) < Tyr (IC_50_ = 571.32 µg/mL). Similar to the O_2_^●−^ scavenging assay, no significant differences (*p* > 0.05) were observed between the positive controls, whilst the results reported for OC and Tyr were significantly different (*p* < 0.05).

The ROO^●^ quenching capacity assay, also called the oxygen radicals absorbance capacity (ORAC) assay, estimates free radical injuries to a fluorescent probe in the presence of antioxidants [24]. Among samples, OC was the most effective ROO^●^ quencher (0.015 µmol TE/mg dw). Significant differences (*p* < 0.05) were observed between the positive controls and the samples. Moreover, OC and Tyr were not significantly different (*p* > 0.05).

### 2.3. Cell Viability Assays

**Figure 2:**
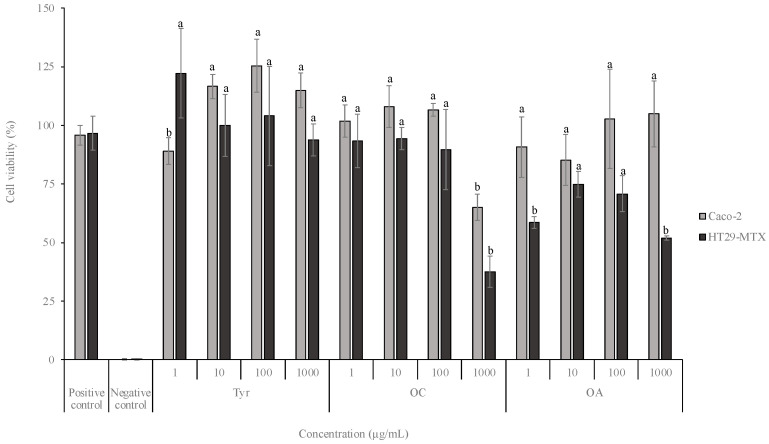
Effects of Tyr, OC, and OA on the viability of Caco-2 and HT29-MTX after exposure to concentrations between 1 and 1000 μg/mL. The values are presented as mean ± standard deviation (*n* = 3). Different letters in the same sample indicate significant differences between concentrations of the same sample (*p* < 0.05).

The effect of the different compounds on the viability of intestinal cells (in concentrations ranging between 1 and 1000 µg/mL), namely, Caco-2 and HT29-MTX, was assessed by an MTT assay (Figure 2).

Concerning HT29-MTX cells, the exposure to Tyr did not lead to a viability decrease for all tested concentrations (*p* > 0.05). Conversely, the exposure to the highest OC concentration tested (1000 µg/mL) conducted to a viability of 37.5%, with significant differences for the other concentrations (*p* < 0.05). Regarding OA, in a general way, the viability results for all tested concentrations were lower, despite none being below 50%.

### 2.4. Intestinal Permeation Assay

**Figure 3:**
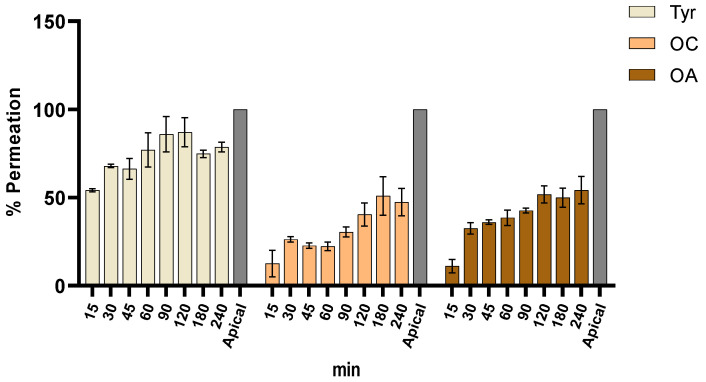
Permeation of Tyr, OC, and OA at 0 min and after 240 min through the intestinal model (*n* = 3).

The intestinal permeation of OC, OA, and Tyr was assessed in an intestinal co-culture model composed by Caco-2 and HT29-MTX at different times, from 0 to 240 min (Figure 3). The permeation results were expressed as percentage of compound release calculated as the ratio between the concentration of the apical side (100%) and the percentage permeated through the model between the apical (t = 0 min) and the basolateral side (t = 240 min). Samples were withdrawn from the receptor side after 15, 30, 60, 90, 120, 150, 180, and 240 min of the apical exposure to determine the bioactive compound transport across the monolayer. As showed in Figure 3, Tyr achieved the highest permeation after 120 min with almost 70%, while OC and OA presented lower permeation values (~50%).

**Figure 4:**
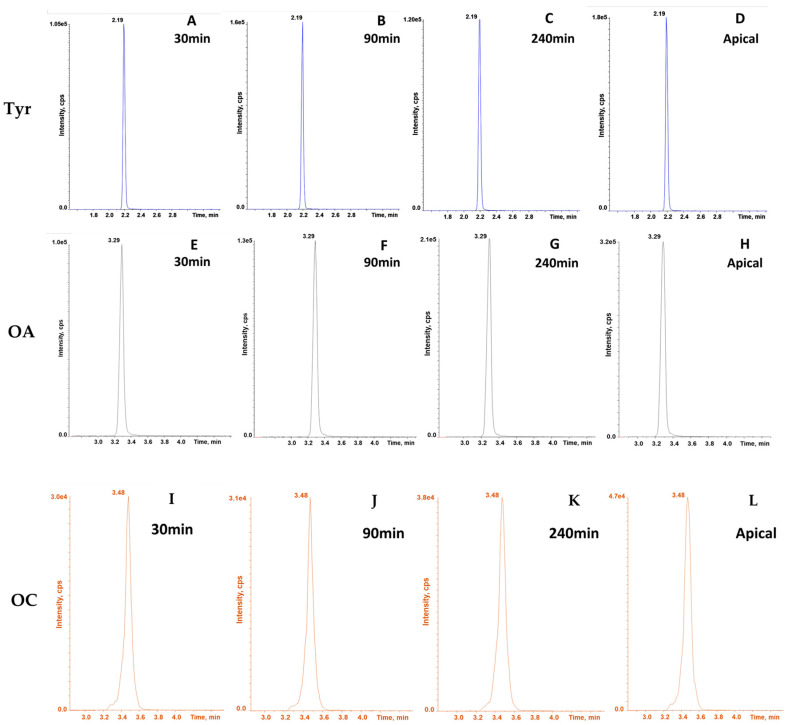
Chromatograms of Tyr OA and OC in representative samples. (**A**–**L**) The intensity of the blue SRM transitions (137.0 → 106.0) shows a linear progression in the permeation of Tyr through the intestinal barrier (**A**–**C**), from apical side (**D**). The intensity of the grey SRM transitions (319.2 → 199.0) shows a linear progression in the permeation of OA through the intestinal barrier (**E**–**G**) from the apical side (**H**). The intensity of the brown SRM transitions (303.1 → 59.0) shows a linear progression in the permeation of OC through the intestinal barrier (**I**–**K**) from the apical side (**L**). Chromatograms were obtained using ABSciex Analyst^®^ software (version 1.7), while data analysis was accomplished using the Microsoft 365^®^ PowerPoint software (Albuquerque, NM, USA). Further MS details regarding the mass spectra of each analyte are reported in Figure S3.

The linear trends of permeation of the OC, Tyr, and OA samples are shown in Figure 4 (panels A–D for Tyr, E–H for OA, and panels I–L for OC). The increase of the intensity of chromatogram peaks at different timepoints during the permeation assay, in representative samples, is established.

Moreover, as highlighted in Figure 4I–K and Figure S1, a linear trend in OC permeation could be clearly observed, validating the permeation assay. The OC stability was further studied in longer time intervals by spiking a blank sample (HBSS buffer) with OC and monitoring the variation of OC chromatogram peak intensity over a 20-day period (Figure S2). Once again, no significant variation was appreciated in short time intervals as considered for the permeation assay, since the completed OC degradation was clearly visible only after matter of days.

**Figure 5:**
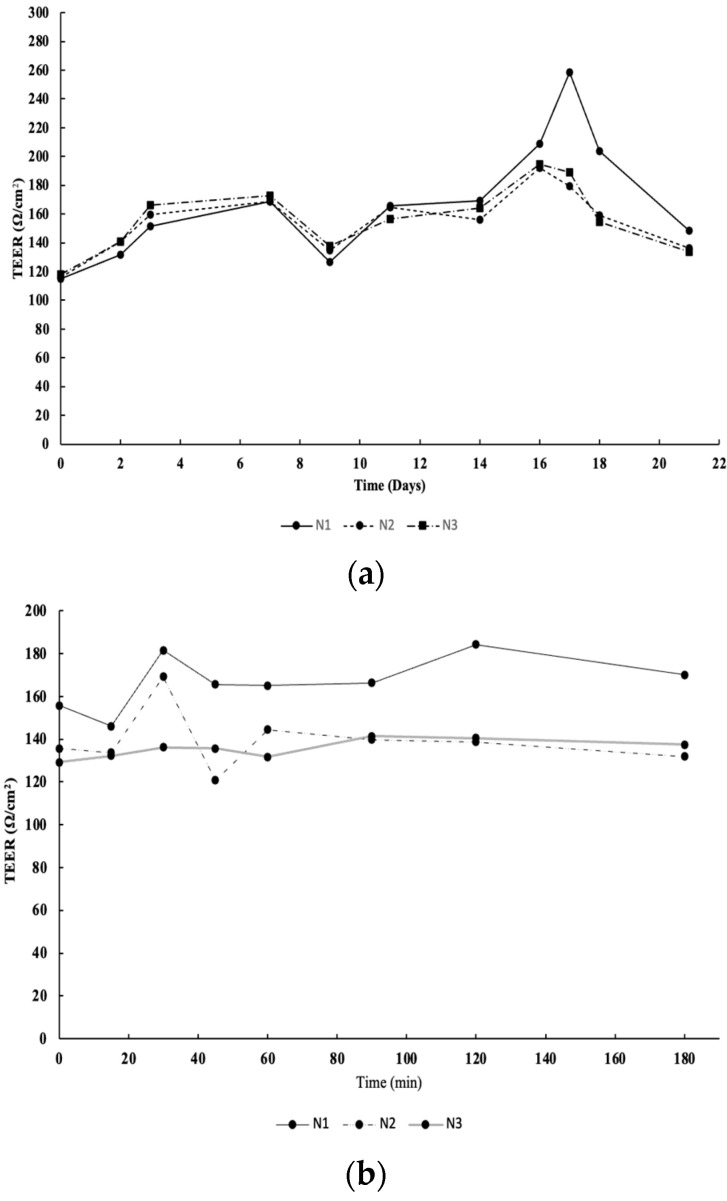
TEER measurements of co-culture cells (90% Caco-2 and 10% HT29-MTX) for 21 days (**a**) as well as during the 3D permeation assay; (**b**) N1, N2, and N3, number of repetitions made.

Figure 5 shows the transepithelial resistance (TEER) values during the 21 days of the model growth (Figure 5a) as well as during the permeation assay (Figure 5b). TEER is a procedure employed to ensure the integrity and permeability of cell cultures, being traditionally used to monitor living cells during growth and differentiation stages [25]. As expected, the values increased until the 14th day, remaining stable until the experiment day (120 ± 20 Ω/cm^2^), which is in line with previous studies [25,26,27,28,29]. The intestinal co-culture model also originates lower TEER values than models only composed by Caco-2 cells, since HT29-MTX cells secret mucus that modulates the Caco-2 tight junctions, leading to inter-cellular spaces.

## 3. Discussion

In recent years, the antioxidant activity of foods has attracted researchers’ interest due to the involvement of ROS in chronic disease onset. The excess of ROS in the human body can promote cumulative damages in DNA, proteins, and lipids, developing the so-called oxidative stress associated with different diseases. This phenomenon is normally described as the imbalance between oxidants and antioxidant agents [30], playing an fundamental role in several diseases, from cardiovascular and neurodegenerative diseases to cancer. For these reasons, antioxidants have a protective role in the human body from negative effects produced by free radicals and ROS. The consumption of nutraceuticals is connected to a reduced mortality due to age-related pathologies, which may be partly endorsed to the presence of hydrophilic antioxidants, especially phenolic compounds [31,32]. In the present work, the antioxidant/antiradical capacities of three important polyphenols, namely Tyr, OC, and OA, were evaluated using various methods to obtain a complete compounds profile. Moreover, the assessment of the scavenging capacity of samples towards biologically relevant reactive species, including O_2_^●−^, HOCl, and ROO^●^, is of the upmost importance to appraise the potential in vivo antiradical activities of OC, Tyr, and OA.

Regarding the DPPH assay, Tyr and OA revealed a low scavenging activity, achieving IC_50_ values > 1 mg/mL, whereas OC showed a better antiradical effect (IC_50_ = 660 ug/mL). Concerning ABTS^●+^, all samples showed the ability to scavenge this radical, with OC presenting an IC_50_ of 70.37 µg/mL, whereas its metabolites Tyr and OA obtained IC_50_ values less than 10 µg/mL.

Based on the FRAP values, the redox potential of all compounds corresponds to 0.09–0.028 µmol of TE/mg dw. As it is possible to observe, OC and Tyr revealed a similar antioxidant profile, whereas OA was two times less potent as a redox antioxidant. All the analyzed polyphenols, particularly OA, despite a good redox in FRAP and ABTS assays, revealed lower IC_50_ values for the DPPH test. These disaccording values in assessing the radical scavenging activity might be related to the nature of the different assays [33].

ROS are described as biological side products of metabolic reactions and include O_2_^●−^ and ROO^●^ as well as H_2_O_2_ and HOCl. These radical species may damage proteins, lipids, carbohydrates, and nucleic acids, often inducing irreversible functional modifications. Moreover, they may interact with DNA, leading to mutations. Consequently, the ROS overproduction of—and the inability of the antioxidant defense system in counteracting—the generated reactive species may conduct to oxidative stress, inducing harmful effects on biomolecules and interfering with metabolic pathways [34]. Therefore, ROS are the major driving causes of oxidative stress-mediated pathologies, such as Alzheimer’s and Parkinson’s diseases, cancer, diabetes, and premature aging [34]. Table 2 summarize the ROS results. Concerning O_2_^●−^ inhibition, the obtained results were substantially higher than the ones documented for ethanolic extracts of EVOOs derived from Picual and Arbequina varieties (12.2% and 7.4% at 10 mg/mL, respectively) [35]. Otherwise, EVOO from a blend of Picual and Arbequina cultivars showed a slightly higher ability to scavenge O_2_^●−^ (21.4% at 10 mg/mL) [35]. In addition, Valavanidis et al. studied the O_2_^●−^ quenching capacity of different vegetable oils from olive, soybean, sunflower, and corn and three phenolic compounds abundant in these oils, namely Tyr, hydroxytyrosol, and oleuropein [36]. According to the authors, the IC_50_ for Tyr (15 µM) was significantly lower than the one obtained in the present study, suggesting a higher O_2_^●−^ scavenging capacity. Conversely, all vegetable oils tested were worst O_2_^●−^ scavengers than Tyr and OC, achieving higher IC_50_ values (25–45 mg/mL) [36]. Regarding the HOCl inhibition assay, OC showed a similar counteracting ability to acetone:water (70:30) and 70% ethanol extracts from *Cytisus scoparius* (IC_50_ of 56.0 and 60.0 µg/mL, respectively), as stated by González et al. [37]. Conversely, Berto et al. reported lower IC_50_ values for ethanolic extracts of *Quararibea cordata* pulp (IC_50_ = 22.0 µg/mL) than the ones obtained for OC and Tyr [36]. Nevertheless, as far as we know, the Tyr and OC quenching capacities against HOCl have not yet been reported, encompassing a new research field. The results of the ROO^●^ quenching capacity assay for Tyr, OC, and OA are higher than the ones reported by Shi et al. for cold pressed kernel oils obtained from different *Eucommia ulmoides* olive cultivars (130.47–243.11 µmol TE/100 g oil) [38]. Nonetheless, the ROO^●^ scavenging capacities of OC and Tyr were even better than the ones achieved for other plant-derived extracts, including *Quercus cerris* kernels and infusions and decoctions from *Actinidia arguta* fruits [39,40]. Furthermore, Sánchez et al. studied the ROO^●^ quenching power of a virgin olive oil obtained in different harvest periods (2001–2004) [41]. The results (1.46–4.97 µmol TE/g oil) were lower than the one obtained for OC in the present study. Oppositely, olive oil exhibited a similar ROO^●^ quenching potential to Tyr [41].

The promising results obtained for the phenolic compounds, including Tyr, regarding in vitro radicals scavenging capacity have been broadly documented in previous studies [42,43]. Noteworthy, this is the first study that investigated the ROS quenching ability of OC. The present study demonstrated the capacity of OC and Tyr as potent scavengers of ROS, namely HOCl and O_2_^●−^.

Aiming a nutraceutical application of the phenolic compounds, a cell viability assay on intestinal cell lines was performed, reporting that the highest concentration of polyphenols tested (1000 µg/mL) led to a viability decrease for both cell lines. To the best of our knowledge, this is the first study that evaluates the effect of OC and OA on intestinal cell lines. Nikou et al. [44] evaluated the effect of OC on human newborn foreskin diploid fibroblasts at different concentrations (50, 100, 150, and 200 µM) and observed that both compounds were relatively non-toxic (threshold of 80%), which is in line with the present study. According to the same authors, OA could promote the cellular antioxidant and antiradical responses by the upregulation of some genes (NQO1, TXNRD1, and KEAP1), probably by activation of NRF2 pathway. Recently, Serreli et al. exposed Caco-2 cells to Tyr and observed the absence of toxicity in concentrations ranging between 0.1 and 10 µM [45]. The authors pre-treated the cells with the pro-inflammatory agent Lipopolysaccharide (LPS) to activate the nitric oxide (NO) production pathway and studied the iNOS expression. The results revealed that Tyr inhibited the NO release, as well as the iNOS expression and the NF-ĸB activation, contributing to preserve the mucosal integrity [17]. Despite these insights on the molecular mechanisms of Tyr, OC, and OA, no studies have explored the compounds potential permeation on an intestinal model, an essential assay to ensure the absorption by humans.

To understand the interaction of the bioactive compounds with the digestive system it is of critical importance to evaluate their pharmacokinetic properties [46]. Therefore, the intestinal permeation was assessed using an in vitro model, aiming to detect the absorbance and the consequent health effect of the compounds. In fact, from a regulatory point of view, the high permeation is intrinsically connected to the fraction of an administered dose that is absorbed. In recent years, several studies were focused on the evaluation of intestinal permeability of bioactive compounds [29,47,48].

The permeation results of OC, OA, and Tyr demonstrated a good permeation for all polyphenols. In general, although OC and OA were able to achieve a permeation of 50% after 240 min, Tyr was the bioactive compound with the best permeation value, affording 78% of release at the final timepoint. These data give important information on the pharmacokinetic profile of the tested polyphenols and, particularly, of OC.

Different aspects could affect the transport of bioactive compounds in the intestine, such as concentration, molecule size and polarity, but also degradation processes. In the literature, only a few studies discussed the pharmacokinetic of OC. For instance, Lopez-Yerena recently reported the in vivo intestinal absorption of OC in rats. In this study, an in-situ perfusion technique was adopted and the OC phase I and phase II metabolism was demonstrated, even without a further characterization of all formed metabolites [49].

OC contains two aldehyde groups (C-1 and C-2), highly electrophilic, able to spontaneously react with other functionality, affording different metabolites and making the OC detection challenging. The highly reactive hydrogens of OC aldehydes render the molecules prone to equilibria and, consequently, more sensitive and unstable.

In the present permeation assay study, performed in HBSS buffer, the LC-MS/MS chromatograms revealed a linear progression in the OC permeation during the experiment (Figure 4I–K). As expected, detectable concentrations of Tyr and OA metabolites were observed, shortly after the beginning of the experiment in the apical side of the OC sample, as a result of possible oxidation and hydrolysis reactions. In fact, similarly to the metabolism of oleacein [50], which produces hydroxytyrosol after hydrolytic phase I metabolism promoted by carboxylesterases, tyrosol was detected in the present study as the main metabolite of OC. Hydrolysis is a common process in phase I drug metabolism, correlated to different mechanisms of hydrolysis reactions. Among them, carboxylesterases (CAE) are enzymes able to catalyse the hydrolysis of esters, amides, and carbamates, generating the corresponding carboxylic acids and alcohols. Moreover, it was possible to observe the formation of OA, probably due to oxidation processes.

## 4. Materials and Methods

### 4.1. Chemicals and Standards

OC and OA as pure standards were isolated from EVOO, following the procedure reported in our previous studies and their characterization by MS analysis (Figure S3), ^1^H NMR and ^13^C NMR (Figures S4 and S5) are reported in the Supplementary Materials [23,51]. Tyr was purchased from TCI Chemicals (Zwijndrecht, Belgium). All reagents and solvents were of high analytical grade, supplied by commercial sources. A,α’-azodiisobutyramidine dihydrochloride (AAPH), dihydrorhodamine 123 (DHR), fluorescein sodium salt, β-nicotinamide adenine dinucleotide (NADH), nitroblue tetrazolium chloride (NBT), phenazine methosulphate (PMS), 2,2′-Azino-bis(3-ethylbenzothiazoline-6-sulfonic acid) diammonium salt (ABTS), 2,2-Diphenyl-1-picrylhydrazyl (DPPH), 2,4,6-Tris(2-pyridyl)-s-triazine (TPTZ), and sodium hypochlorite solution with 4% available chlorine were acquired from Sigma-Aldrich (Steinheim, Germany), as well as catechin, gallic acid, and Trolox**^®^**. Cell reagents were provided by Life Technologies, S.A. (Madrid, Spain) and Invitrogen Corporation (Life Technologies, S.A., Madrid, Spain). Caco-2 clone type C2BBe1 and HT29-MTX cell lines were acquired from the American Type Culture Collection (ATCC, Manassas, VA, USA).

### 4.2. Reactive Oxygen Species Scavenging Capacity Assays

A Synergy HT Microplate Reader (BioTek Instruments, Inc., Winooski, VT, USA) equipped with a thermostat was used to estimate the quenching capacity of Tyr and OC against reacting oxygen species (ROS). Catechin and gallic acid were used as positive controls. Following the procedure described by Pinto et al., samples and positive controls were dissolved in phosphate buffer [24]. Three independent experiments (*n* = 3) were carried out for each assay using six concentrations. The curves of inhibition percentage versus antioxidants concentration were plotted using GraphPad Prism 7 software (La Jolla, CA, USA). The ROS scavenging capacity of OA has been evaluated in our previous paper [23].

#### 4.2.1. Superoxide Anion Radical Scavenging Assay

The superoxide anion radical (O_2_^●−^) scavenging assay was performed using the method designed by Gomes et al. [52], as described in the Supplementary Materials. Results were presented as the inhibition, in percentage or IC_50_ (µg/mL), of the NBT reduction to diformazan.

#### 4.2.2. Hypochlorous Acid Scavenging Assay

According to Gomes et al. [52], the hypochlorous acid (HOCl) scavenging capacities of samples and positive controls were determined using a HOCl solution prepared with 1% (*w*/*v*) NaOCl at pH 6.2, as described in the Supplementary Materials. Results were expressed as the inhibition, in IC_50_ (µg/mL), of the HOCl-induced oxidation of DHR to rhodamine.

#### 4.2.3. Peroxyl Radical Scavenging Assay

Following the methodology validated by Ou et al. [53], the quenching abilities of the tested compounds and positive controls against peroxyl radical (ROO^●^) were assessed by an oxygen radical absorbance capacity (ORAC) assay. Trolox**^®^** was used as standard. The results were expressed as µmol of Trolox**^®^** equivalents (TE) per mg of sample on dry weight (dw).

#### 4.2.4. 2,2-Diphenyl-1-picrylhydrazyl (DPPH) Assay

The antiradical activities of Tyr, OC, and OA were evaluated by the free radical scavenging of DPPH, using the protocol reported by Brand–Willians [54], with some modifications [55], as described in the Supplementary Materials. Methanol was used as the blank, Trolox^®^ was employed as the positive control, and DPPH^•^ solution as the negative control. The results were expressed as an inhibitory concentration at 50% (IC_50_). All experiments were performed in triplicate.

#### 4.2.5. ABTS Assay

The free radical scavenging activity of samples was determined by ABTS radical cation decolouration assay using the protocol reported by Pellegrini et al. with some modifications [55,56,57], as described in Supplementary Materials. Trolox^®^ was employed as the positive control. The percentage of scavenging ability was calculated against sample concentration to obtain the inhibitory concentration at 50% (IC_50_). All experiments were performed in triplicate.

#### 4.2.6. Ferric Reducing/Antioxidant Power (FRAP) Assay

The method described by Borges et al. with some modifications was used to evaluate the antioxidant activity of Tyr, OC, and OA [55,58], as described in Supplementary Materials. The calibration curve was constructed using different concentrations of Trolox**^®^** (0.01–0.2 mg mL^−1^) and the results were expressed as µmoles of Trolox**^®^** equivalents per milligram of sample in dry weight (µmol TE/mg dw). All experiments were performed in triplicate.

### 4.3. Cell Viability Assay

The cell viability after exposure to the samples was evaluated by a 3-(4,5-dimethylthiazol-2-yl)-2,5-diphenyltetrazolium bromide (MTT) assay. For this purpose, Caco-2 (passages 81–84) and HT29-MTX (passages 35–38) cell lines were used. The cell viability assay was performed according to Silva et al. [25,26] after exposing the cells to the samples at different concentrations (0.1, 1, 10, 100, and 1000 µg/mL). The positive control used was DMEM and the negative control was 1% (*w*/*v*) Triton X-100. Results were expressed as percentages (%) of cell viability.

### 4.4. Intestinal Permeability Assay

The intestinal model, composed of a co-culture of Caco-2 and HT29-MTX cell lines, was prepared according to the original model developed and validated by Araújo and Sarmento, and slight modified by González et al. [29]. The experiments were performed 21 days after seeding the cells in 12-well plates. During this period, the transepithelial electrical resistance (TEER) was monitored to evaluate the cell monolayer integrity. On the last day, cell monolayers were pre-equilibrated with fresh HBSS (pH 7.4) at 37 °C for 30 min. Afterwards, 0.5 mL of the sample (500 µg/mL) prepared in HBSS was added to the apical side of the co-culture monolayers and 1.5 mL of HBSS to the basolateral side. Samples were withdrawn from the receptor side at different timepoints (0, 15, 30, 45, 60, 90, 120, 150, 180, and 240 min) to determine the bioactive compounds transported across the monolayer. At the same time, the TEER was evaluated, employing an EVOM Epithelial Volthometer Instrument equipped with a chopstick electrode (World Precision Instruments, Sarasota, FL, USA). After each sampling time, the basolateral side was replaced with the same HBSS volume. Samples were conserved at −20 °C for subsequent LC/ESI-MS analysis, according to Section 4.5. The transepithelial electrical resistance (TEER) was quantified before, during, and at the end of the assay.

### 4.5. LC-MS/MS Analysis

The instrument layout consisted of an Agilent (Santa Clara, CA, USA) 1290 UHPLC system including a column oven set at 40 °C, a binary pump, and a thermostated autosampler coupled to an AB-Sciex (Concord, ON, Canada) QTRAP 6500+ mass spectrometer working as a triple quadrupole, equipped with an IonDrive™ Turbo V source. Separation was chromatographically achieved with a 2.1 × 50 mm, 1.7 μm particle size, Acquity UPLC BEH Phenyl column (Waters Corporation, Milford, MA, USA), protected by a Acquity UPLC BEH Phenyl VanGuard Pre-Column and using MeOH 100% (A) and water 100% (B) as mobile phases. Gradient elution (0.6 mL/min) was performed as follows: 0.0–1.0 min 100% B; 4.5–5.5 min 5% B; 6.5–7.5 min 100% B. The volume of injection was set at 5 μL. Data acquisition and system control were performed using the ABSciex Analyst**^®^** software (version 1.7), while data analysis was accomplished using the Microsoft 365**^®^** Excel software (Albuquerque, NM, USA). A selected reaction monitoring (SRM) mass spectrometry method was operated in negative ion mode. For each analyte, after optimizing the declustering potential (DP), collision energy (CE), and collision exit potential (CxP), three transitions were accounted in the analysis. One of them was integrated and used as a quantifier trace (Q) while the other two were used as qualifier traces (q), as reported in Table 3. Additional operative parameters were ion spray voltage (ISV), −2.25 kV, gas source 1 (GS1), 20 arbitrary units (au); gas source 2 (GS2), 40 au; Curtain gas (CUR), 20 au; temperature of the source (TEM), 500 °C; collision gas (CAD) N_2_, operative pressure with CAD gas on, 2.3 mPa; and entrance potential (EP), −7V.

The analytes were quantified by calibration curves, prepared daily in water solutions by serial dilution of standards at concentrations ranging from 0.97 to 1000 ng/mL. Samples, kept at −80 °C, were thawed at room temperature before analysis, diluted in water and directly injected into the LC-MS/MS system.

### 4.6. Statistical Analysis

Data were presented as mean ± standard error of three independent experiments. IBM SPSS Statistics 24.0 software (SPSS Inc., Chicago, IL, USA) was employed to evaluate statistical differences among results. A one-way analysis of variance (ANOVA) was applied to determine the differences between samples and post hoc comparisons of the means were carried out using Tukey’s HSD test. A denoting significance was accepted for *p* < 0.05.

## 5. Conclusions

Nowadays, the beneficial properties of extra virgin olive oil are widely studied, recognizing the fundamental role of polyphenols as nutraceutical resource. The most representative polyphenols in EVOO are simple phenols such as Tyr and hydroxytyrosol and the secoriroid oleacein and OC. Because they are responsible for the majority of the health effects of EVOO, the study of nutraceutical properties and metabolism of EVOO’s principal polyphenols is crucial.

In the present study, the antioxidant and antiradical activities of Tyr, OC, and OA were investigated with the aim to validate their potential use as nutraceutical ingredients. The capacity of OC and Tyr as potent ROS scavengers, particularly regarding HOCl and O_2_^●−^, was clearly demonstrated. Furthermore, the ability to permeate the intestinal membrane was assessed by an intestinal co-culture model composed by Caco-2 and HT29-MTX cell lines. The permeation results of OC, OA, and Tyr demonstrated a good permeation for all polyphenols. In general, although OC and OA were able to achieve a permeation of 50% after 240 min, Tyr was the bioactive compound with the best permeation value, affording 78% of release at the final timepoint. Further studies, focused on in vivo assays, should be performed to attest the in vitro results obtained, as it is important to guarantee the safety and efficacy of these new nutraceutical ingredients.

## Figures and Tables

**Table 1:** Antiradical and antioxidant activities of OC, Tyr, and OA evaluated, respectively, by ABTS, DPPH, and FRAP assays. Values are expressed as mean ± standard error of the mean (*n* = 3).

	FRAP	DPPH	ABTS
	µmol TE/mg dw	IC_50_ (µg/mL)	IC_50_ (µg/mL)
OC	0.021 ± 0.002 ^a^	660 ± 26 ^b^	70.37 ± 2.02 ^a^
Tyr	0.028 ± 0.0009 ^a^	1060 ± 47 ^a^	2.17 ± 0.07 ^c^
OA	0.009 ± 0.002 ^b^	>2000	9.91 ± 1.05 ^b^

IC_50_ = in vitro concentration required to decrease by 50% the reactivity of the reactive species in the tested media (mean ± standard error of the mean). Different letters (^a, b, c^) in the same column indicate significant differences between samples (*p* < 0.05).

**Table 2:** Superoxide anion radical (O_2_^●−^), hypochlorous acid (HOCl) and peroxyl radical (ROO^●^) scavenging capacities of Tyr and OC (OA values reported previously) [23]. Values are expressed as mean ± standard error of the mean (*n* = 3).

	Reactive Oxygen Species			
	O_2_^●−^		HOCl	ROO^●^
	IC_50_ (µg/mL)	% Inhibition	IC_50_ (µg/mL)	µmol TE/mg dw
OC	919.80 ± 34.30 ^a^	-	73.18 ± 1.43 ^b^	0.0152 ± 0.0029 ^c^
Tyr	-	17.05 ± 0.67	571.32 ± 8.50 ^a^	0.0046 ± 0.0007 ^c^
OA		19.09 ± 1.20 ^a,^*	360.87 ± 8.79 ^a,^*	0.0056 ± 0.0003 ^a,^*
Positive controls				
Catechin	48.05 ± 0.78 ^b^	-	0.22 ± 0.01 ^c^	0.44 ± 0.07 ^b^
Gallic acid	12.04 ± 0.03 ^b^	-	4.80 ± 0.06 ^c^	1.39 ± 0.11 ^a^

IC_50_ = in vitro concentration required to decrease by 50% the reactivity of the reactive species in the tested media (mean ± standard error of the mean). Different letters (^a, b, c^) in the same column indicate significant differences between samples (*p* < 0.05). * Data previously reported [23].

**Table 3:** MS operative parameters for Tyr, OC, and OA.

Analyte	SRM Transition (Da)	DP (V)	CE (V)	CxP (V)
Tyr	137.0 → 106.0 (Q)		−20	−5.0
	137.0 → 107.0 (q)	−35	−21	−5.4
	137.0 → 119.0 (q)		−20	−5.9
OC	303.1 → 59.0 (Q)		−10	−6.0
	303.1 → 165.0 (q)	−30	−13	−8.4
	303.1 → 182.6 (q)		−13	−9.6
OA	319.2 → 110.8 (q)		−23	−5.3
	319.2 → 155.0 (q)	−25	−25	−7.5
	319.2 → 199.0 (Q)		−19	−9.8

## Data Availability

Not applicable.

## Supplementary Materials

The following supporting information can be downloaded at: https://www.mdpi.com/article/10.3390/molecules28135150/s1, Figure S1. OC time dependent trend. Figure S2. OC Stability test. Figure S3. Significative chromatograms and mass spectra of OC, Tyr and OA in representative samples. Figure S4A 1H NMR (400 MHz, CDCl3) and Figure S4B 13C NMR (100 MHz, CDCl3) of OC.; Figure S5A 1H NMR (400 MHz, CDCl3) and Figure S5B 13C NMR (100 MHz, CDCl_3_) of OA.

## Sample Availability

Samples of the compounds are available from the authors.
