# Supplementary material for: Insights into the Antioxidant/Antiradical Effects and In Vitro Intestinal Permeation of Oleocanthal and Its Metabolites Tyrosol and Oleocanthalic Acid
Source: Molecules. 2023 Jun 30;28(13):5150. doi: 10.3390/molecules28135150 (PMC10343523; doi:10.3390/molecules28135150)
Supplement: Supplementary file 1 [file molecules-28-05150-s001.zip › molecules-2464494-supplementary.pdf]

SUPPLEMENTARY INFORMATION

Insights into the Antioxidant/Antiradical Effects and In Vitro Intestinal Permeation of Oleocanthal and Its Metabolites Tyrosol and Oleocanthalic Acid

Doretta Cuffaro [1,2], Diana Pinto [3], Ana Margarida Silva [3], Andrea Bertolini [4], Simone Bertini [1], Alessandro Saba [4], Marco Macchia [1,2], Francisca Rodrigues [3,*] and Maria Digiacomo [1,2,*]

[1] Department of Pharmacy, University of Pisa, 56126 Pisa, Italy; doretta.cuffaro@unipi.it (D.C.); simone.bertini@unipi.it (S.B.); marco.macchia@unipi.it (M.M.)

[2] Interdepartmental Research Center "Nutraceuticals and Food for Health", University of Pisa, 56100 Pisa, Italy

[3] REQUIMTE/LAQV, ISEP, Polytechnique Institute of Porto, Rua Dr. António Bernardino de Almeida 431, 4200-072 Porto, Portugal; diana.pinto@graq.isep.ipp.pt (D.P.); ana.silva@graq.isep.ipp.pt (A.M.S.)

[4] Department of Surgery, Medical, Molecular and Critical Area Pathology, University of Pisa, 56126 Pisa, Italy; a.bertolini2@student.unisi.it (A.B.); alessandro.saba@unipi.it (A.S.)

* Correspondence: francisca.rodrigues@graq.isep.ipp.pt (F.R.); maria.digiacomo@unipi.it (M.D.)

1. Experimental section

1.1 2,2-Diphenyl-1-picrylhydrazyl (DPPH) assay

The antiradical activity of Tyr, OC and OA was evaluated by the free radical scavenging of DPPH, using the protocol reported by Cuffaro et al.[1]. Briefly, 100 µL of DPPH solution in methanol (40.0 µg/mL) was added to 100 µL of sample solution in methanol (concentration range: Tyr 1.5- 0.1 mg/mL, OC 680-9.5 µg/mL, OA 2 – 0.2 mg/mL). After 45 min of incubation at room temperature and in the dark, the absorbance was read at 517 nm in a Molecular Devics SPECTROstarNano (200–1000 nm) UV/Vis spectrophotometer. Methanol was used as blank, and $DPPH^{\bullet}$ solution as negative control. Trolox® was employed as positive control and was treated under the same conditions as the samples using a different concentration range (10-0.5 µg/mL, IC_{50}= 6.8 µg/mL). The percent of antioxidant activity (%AA) was calculated according to the following equation:

$$\%AA = ((Abs_{DPPH}) - (Abs_{sample})/Abs_{DPPH}) \times 100$$

Abs_{DPPH}= the absorbance of the DPPH solution

Abs_{sample}= the absorbance of the DPPH solution containing the test compound.

The results were expressed as inhibitory concentration of 50% (IC_{50}). All experiments were performed in triplicate.

1.2 ABTS assay

The free radical scavenging activity of samples was determined by ABTS radical cation decolouration assay using the protocol reported by by Cuffaro et al. [1] Briefly, the ABTS solution was prepared mixing 7 mM of aqueous solution of ABTS with 2.45 mM potassium persulfate in a 1:1 ratio. The solution was incubated for 12h in the dark at room temperature and, afterwards, diluted with ethanol to obtain an absorbance of 0.7 at 750 nm. 180 µL of ABTS solution were mixed with 10 µL of sample in ethanol (concentration range: Tyr 2.5-0.375 µg/mL, OC 100-6.25 µg/mL, OA 48-0.8 µg /mL) and the solution was incubated 5 min at room temperature; then, the final absorbance was read at 734 nm. The % scavenging ability was calculated as follow:

$$\% \text{ scavenging ability} = ((Abs_{ABTS}) - (Abs_{sample})/Abs_{ABTS}) \times 100$$

Abs_{ABTS}= the absorbance of the ABTS solution

Abs_{sample}= the absorbance of the ABTS solution containing the test compound.

The percentage of scavenging ability was calculated against the sample concentration to obtain the inhibitory concentration at 50% (IC_{50}). Trolox® was employed as positive control and was treated under the same conditions as the samples using a different concentration range (100-3 μg/mL, IC_{50}= 13.4 μg/mL). All experiments were performed in triplicate.

1.3 Ferric reducing/antioxidant power (FRAP) assay

The method described by Cuffaro *et al.*, with some modifications, was used to evaluate the antioxidant activity of Tyr, OC and OA.[1] The FRAP reagent was prepared mixing 0.3 M acetate sodium buffer pH=3.6, 20 mM ferric chloride and 10 mM TPTZ in 40 mM HCl in ratio 10:1:1. 20 μL of extracts (1 mg/mL) were mixed with 280 μL of FRAP solution. The resulting solution was incubated at 37 °C for 30 min. The absorbance of the reaction mixture was read at 595 nm in a SPECTROstarNano (200–1000 nm) UV/Vis spectrophotometer using the FRAP solution as blank. The calibration curve was constructed using different concentrations of Trolox® (0.01–0.2 mg/mL), and the results were expressed as μmoles of Trolox® per milligram of sample in dry weight (μmol TE/mg dw).

1.4 Reactive oxygen species (ROS)

1.4.1 Superoxide radical scavenging assay

Superoxide radical was generated by the NADH/PMS system and the $O_2^{\bullet -}$ scavenging activity was determined by monitoring the effect of the tested compound on the $O_2^{\bullet -}$ induced reduction of NBT at 560 nm after 2 min. The antioxidant tiron and ascorbic acid were used as positive control. The results were expressed as the inhibition (in percentage) of the NBT reduction to diformazan.

1.4.2 Hypochlorous acid scavenging assay

The HOCl was measured by using a fluorescent methodology. Based on the HOCl-induced oxidation of DHR to rhodamine. HOCl was prepared by adjusting the pH of a 1% (*m/v*) solution of NaOCl to 6.2 with addition of H_2SO_4 (10%). Quercetin and ascorbic acid were used as positive control. The results were expressed as the inhibition (in percentage) of HOCl-induced oxidation of DHR.

Figure S1. OC time dependent trend. Figure S1 summarizes the time dependent trend in OC samples (in brown) revealing a proportional increment in OC, demonstrating its stability in all short time intervals considered for the permeation assay. This is important because this OC increasing trend would not have been observed if the half-life of the molecule was shorter than the time considered to perform the assay. Results were obtained using ABSciex Analyst® software (version 1.7), whereas data analysis was accomplished using the Microsoft 365® PowerPoint software (Albuquerque, New Mexico, USA) and GraphPad Prism version 9.0.

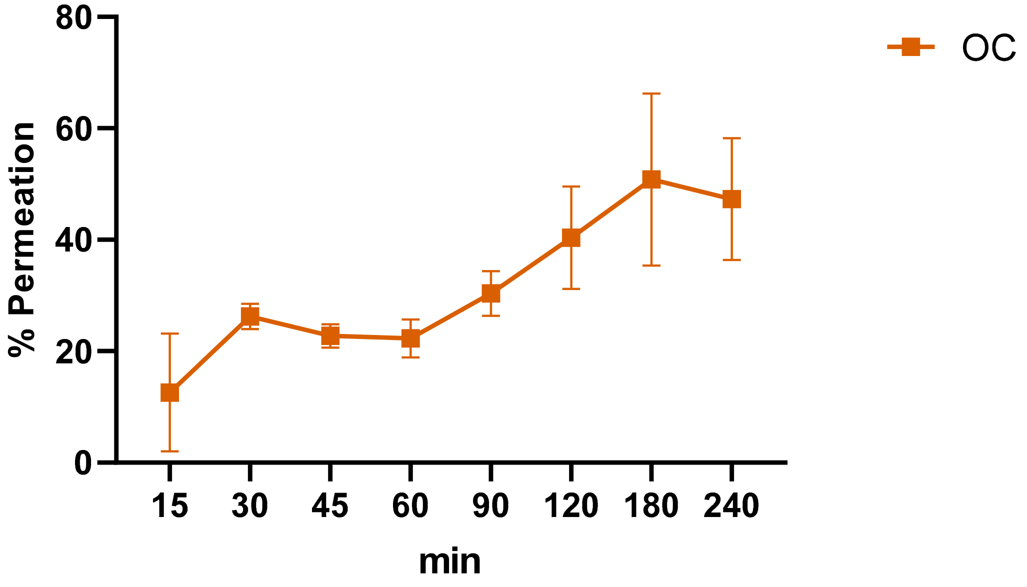

Figure S2. OC Stability test. (**A**–**E**) The brown SRM transitions (303.1 → 59.0) shows the curve stability of OC over a large period of time (0 min to 20 days): OC, spiked in a blank sample (HBSS buffer) at RT, seems to be stable at short intervals of time while its degradation occurs in a matter of days, as shown in panel (**F**), thus confirming the validity of the permeability assay. Chromatograms were obtained using ABSciex Analyst® software (version 1.7), while data analysis was accomplished using the Microsoft 365® PowerPoint software (Albuquerque, New Mexico, USA) and GraphPad Prism version 9.0.

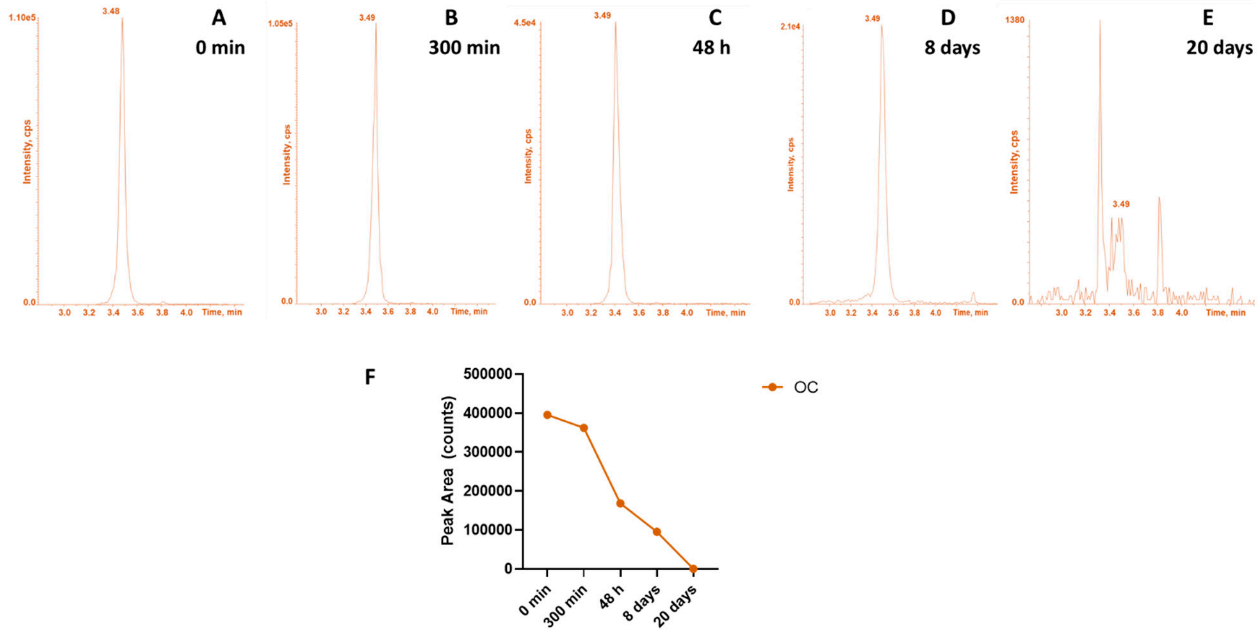

Figure S3. Significative chromatograms and mass spectra of OC, Tyr and OA in representative samples. Panel A shows the chromatogram of OC and the relative spectra of the molecular ion (Q1 MS scan type) and the fragment ions (MS2 Product ion scan type). Panel B shows the chromatogram of Tyr and the relative spectra of the molecular ion (Q1 MS scan type) and the fragment ions (MS2 Product ion scan type). Panel C shows the chromatogram of OA and the relative spectra of the molecular ion (Q1 MS scan type) and the fragment ions (MS2 Product ion scan type). Chromatograms were obtained using ABSciex Analyst® software (version 1.7).

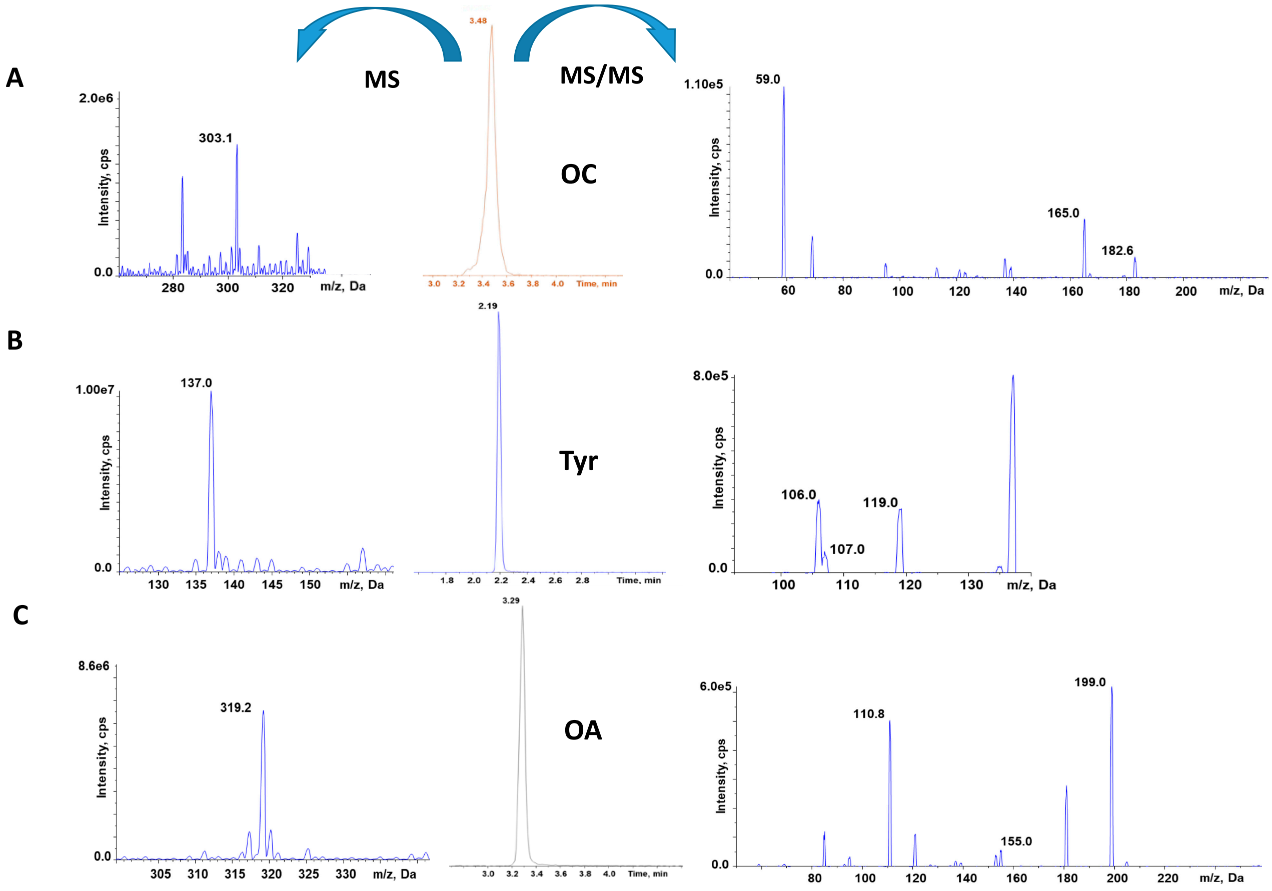

Figure S4- (**A**) ^{1}H NMR (400 MHz, $CDCl_3$) and (**B**) ^{13}C NMR (100 MHz, $CDCl_3$) of OC.

A

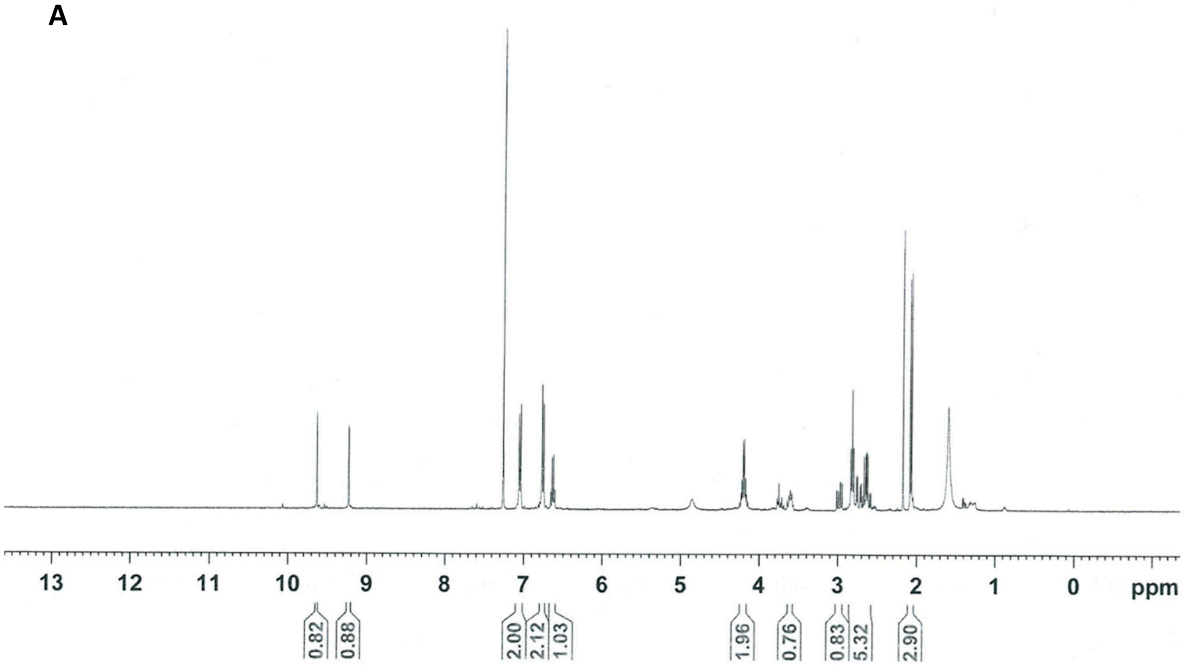

B

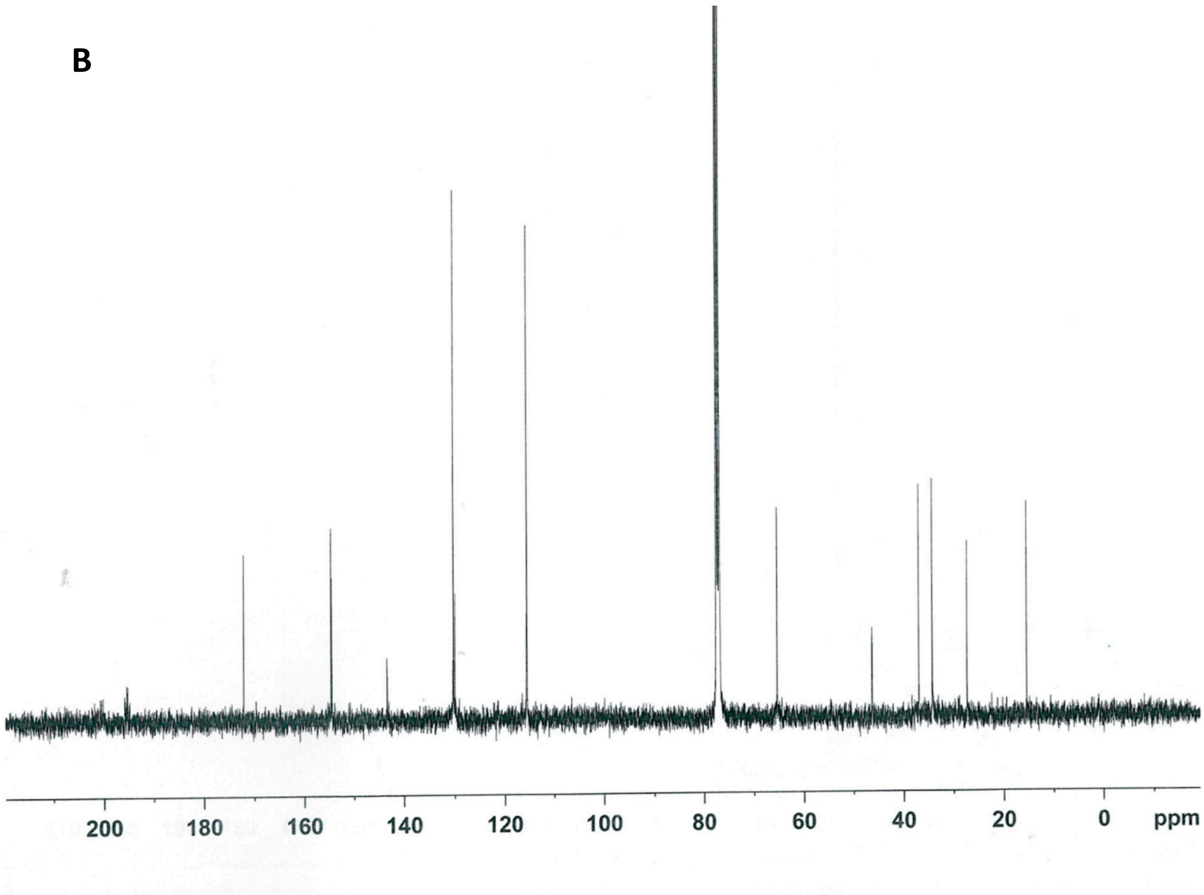

Figure S5- (**A**) ^{1}H NMR (400 MHz, $CDCl_3$) and (**B**) ^{13}C NMR (100 MHz, $CDCl_3$) of OA.

A

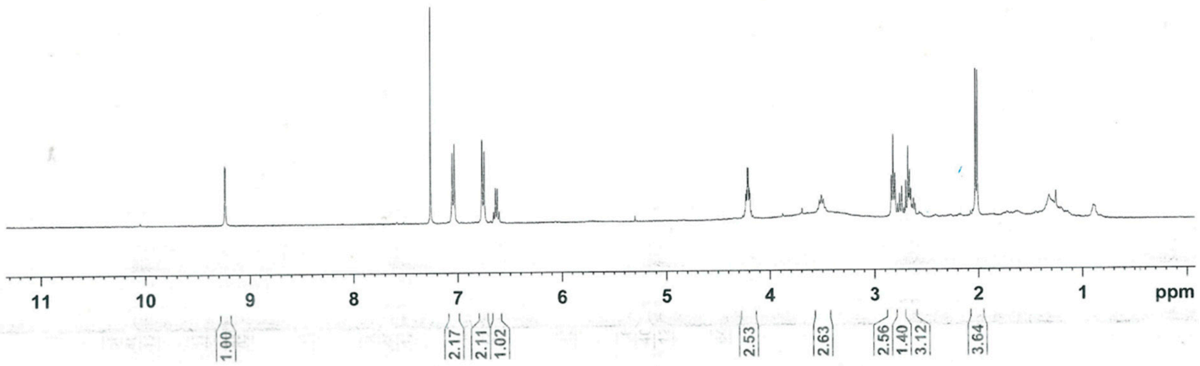

B

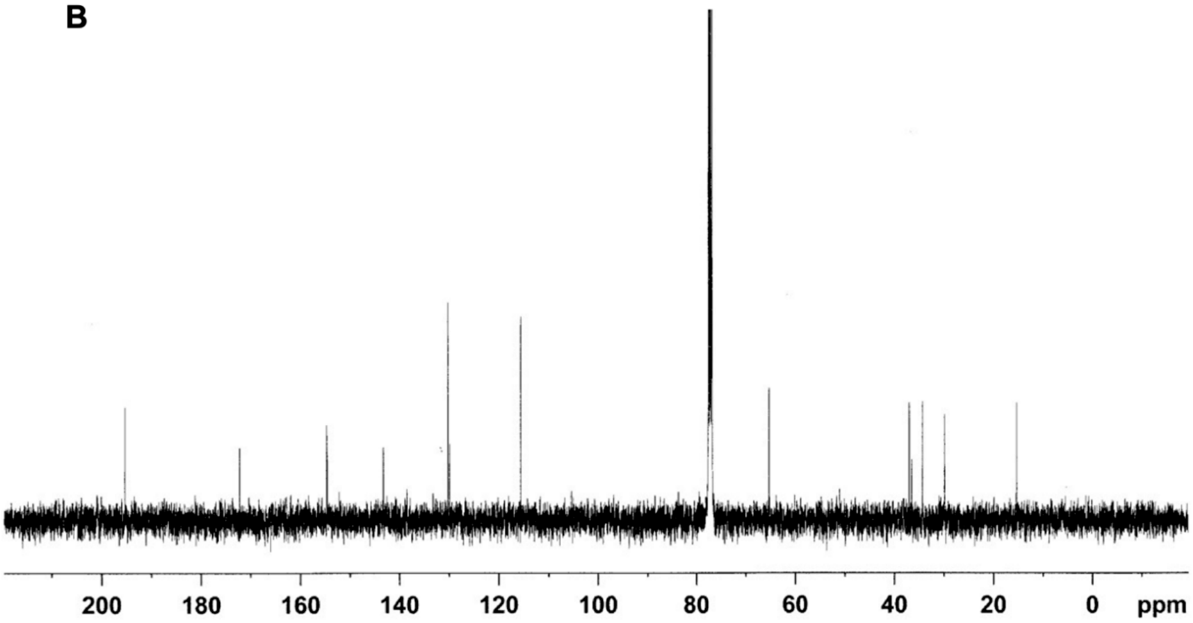

References
1 Cuffaro D, Bertini S, Macchia M, Digiacomo M. Enhanced Nutraceutical Properties of Extra Virgin Olive Oil Extract by Olive Leaf Enrichment. Nutrients. 2023 Feb 21;15(5):1073.
